# Comparison of the anti-reflux ileum valve-pouch orthotopic neobladder and the Studer technique after radical cystecomy: surgical and renal functional outcomes

**DOI:** 10.1186/s12957-025-03699-0

**Published:** 2025-10-02

**Authors:** Zaisheng Zhu, Yiyi Zhu, Wenmin Ying, Han Wu, Penfei Zhou, Quanqi Liu, Jianyong Tong, Yueping Wang

**Affiliations:** 1 Department of Urology, Jinhua HospitalAffiliated to, Zhejiang University School of Medicine, 365 East Renming Road, Jinhua, Zhejiang 321000 China; 2 Department of Endocrinology, Affiliated Second Hospital to Zhejiang University School of Medicine, Hangzhou, 310009 China; 3 Department of Urology, Jinhua Traditional Chinese Hospitalaffiliated, Zhejiang University of Traditional Chinese Medicine , Jinhua, 321000 China

**Keywords:** Bladder cancer, Radical cystectomy, Urinary diversion, Orthotopic neobladder, Propensity score matching

## Abstract

**Background:**

This study describes the construction of an anti-reflux neobladder using an ileum valve-pouch (IVP) and compares its efficacy with that of the modified Studer-pouch (MSP).

**Methods:**

This study included a total of 127 patients who underwent radical cystectomy + neobladder construction (IVP: *n* = 66; MSP = 61) between January 2015 and June 2023 at two major medical centers in our city. Potential bias was reduced by 1:1 propensity score matching (PSM) to compare oncology, complications, and renal function protection between the two groups. The estimated glomerular filtration rate (eGFR) was calculated using the CKD-EPI (Chronic Kidney Disease Epidemiology Collaboration) equation. Survival was assessed using Kaplan–Meier analysis.

**Results:**

The median follow-up time was 44.5 and 35.5 months in the IVP and MSP groups, respectively. After propensity scoring, 84 patients (42 in each group) were included in the analysis.There was no significant statistical difference in the operation time(*p* = 0.128) and the time of urinary diversion (*p* = 0.354) between the two groups.Kaplan–Meier curves showed no significant differences in cancer-specific survival (CSS) (*p* = 0.181) and overall survival (OS) (*p* = 0.611) between the two groups. In addition, the total complications and renal function were not statistically different between the two groups (*p* > 0.05). The incidence of patients who needed to be re-hospitalized due to urinary tract infection was lower in the VIP group than in the MSP group (*p* = 0.039). At 12 months postoperatively, lower rates of decreased eGFR and renal function damage were observed in the IVP group compared to the MSP group (*p* = 0.031 and < 0.001), which were significantly related to the type of neobladder (*p* = 0.004) and preoperative eGFR values (*p* < 0.001).

**Conclusion:**

The preliminary results of VIP technique are safe and effective. It does not increase the time of anti-reflux construction. The incidence of complications similar in the two groups. However, the protection of renal function at 12 months after surgery seemed to be superior to MSP.The independent factors affecting renal function damage are neobladder type and preoperative eGFR.

## Introduction

Radical cystectomy (RC) is the standard treatment for muscle invasive and very high-risk or high-risk non-muscle invasive bladder cancers. Orthotopic neobladder (ONB) substitution significantly improves the quality of life for patients, and has emerged as a commonly used method for urinary diversion [1, 2]. Ureteroenteric anastomosis (UEA) is a crucial step in the construction of ONB, securing the safety of the upper urinary tract (UUT), which is achieved using either reflux or anti-reflux (AR) techniques. AR anastomosis is recommended due to reasons such as non-low pressure in the reservoir, if not resistant to reflux, it is prone to cause greater likelihood of UUT bacterial settlement and susceptibility to pyelonephritis [2–5]. However, AR entails a higher risk of anastomotic stricture compared to the reflux anastomosis technique [6–9]. Hence, whether the AR technique should be employed remains controversial [4, 10–12].The ileum is approximately 20–25 cm long and represents a potential input arm for AR in protecting the UUT; this technique was first reported by Studer in 1991 [13]. With subsequent research, the length of this input arm has been shortened [14]. The present study summarizes the surgical experience ONB over the past 15 years, combining the concept of AR anastomosis of biliary and intestines in general surgery. Ultimately,since 2009, an innovative UEA technique (i.e., Anti-reflux ileal valveplasty and ureteral drag into ileal anastomosis) [15]was independently designed.Two groups were established, one using this technique and the other using the modified Studer neobladder technique [14, 16]. The patients were prospectively recruited and followed up from 2015. This study reports on the technique and its efficacy.

## Patients and methods

### Basic Patient Information

From January 2015 to June 2023, a total of 156 patients who underwent RC + ONB for bladder cancer were recruited from two major medical centers(Jinhua Hospital Affiliated to Zhejiang University School of Medicine and Jinhua Traditional Chinese Hospital affiliated to Zhejiang University of Traditional Chinese Medicine). Among the patients, 2 cases had incomplete medical records, 12 cases had a follow-up of less than 12 months, and 15 cases were excluded as out-of-province patients who did not receive satisfactory follow-up. The remaining 127 patients were included in the analysis. They were divided into two groups according to the RA technique used. Group 1 received anti-reflux ileum valvuloplasty and ureteral drag into ileal anastomosis technique (ileum valve-pouch, IVP) [15], with 66 cases, and group 2 was the modified Studer group with direct anastomosis of ureteral and ileal Nesbit's method (Modified Studer-Pouch,MSP) [14, 16] comprising 61 cases.

Inclusion criteria: having (T2-4N0M0) bladder cancer, negative bladder neck and in good health. Exclusion criteria: patients with serum creatinine greater than 135.0 μmol/L (normal values are 40.0–135.0 μmol/L), inflammatory bowel disease, mental or physical impairment resulting in the inability to self-catheterize when necessary, and conditions such as urethral sphincter abnormalities or urethral strictures.

Propensity score-matching (PSM) was performed to minimize the effects of confounding factors such as the preferences of the primary surgeon and different baseline characteristics of the patients (e.g. age, comorbidities, and tumor risk) [17, 18]. Therefore, PSM was conducted to reduce or eliminate these biases. PSM was calculated using a multivariate logistic regression model for each patient's propensity score, including age, gender,body mass index(BMI), smoking, comorbidities, preoperative hydronephrosis/ureteral obstruction, preoperative renal function, anesthesia ASA (American Society of Anesthesiologists, ASA) risk classification, surgical approach (open/laparoscopic), nerve-sparing(yes/no), pathologic stage, pathologic grade, number of lymph nodes removed, combined carcinoma in situ, abnormal pathology (adenocarcinoma and squamous carcinoma, etc.), perioperative chemotherapy, and duration of follow-up. The propensity score matching ratio was 1:1, and the matching tolerance was 0.02. The study protocol was approved by the Ethics Committee of Jinhua Hospital (ethics number: 2019-ethics-121). All patients signed an informed consent for surgery.

The choice of the surgical procedure for the orthotopic neobladder was determined according to the surgeon's own familiarity and experience preference.This group mainly has 3 attending physicians (ZHU, HU and YING) involved in the surgery. However, all surgeries are by an experienced senior attending physician (ZHU) or under his supervision. The decision of whether to use the orthotopic neobladder (ONB) substitution is made based on a comprehensive assessment of various clinical indicators and surgical plans formulated according to principle.

### Surgical techniques

**Fig. 1:**
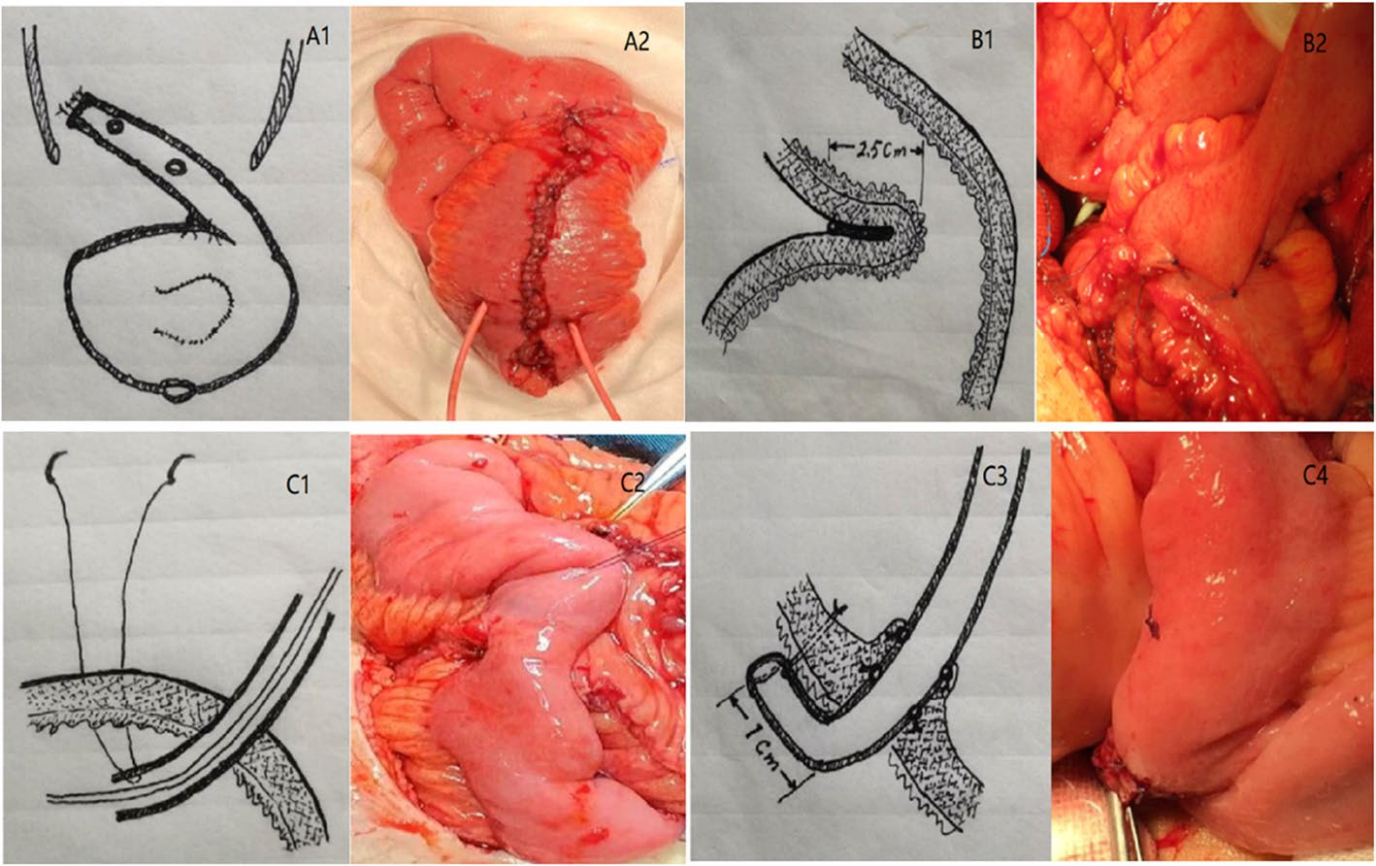
Anti-reflux ileal valveplasty and ureteral drag into ileal anastomosis technique. A1-A2. Shape of the pouch, ileum valve, and ureteral anastomosis site. B1-B2. Anti-reflux ileum valvuloplasty. C1-C2. Ureteral drag into ileal, C3-C4.Double-row fixation of the ureter to the seruscular layer of the ileum with 4-6 stitches. Note: A1/B1/C1/C3 are schematic diagrams, and A2/B2/C2/C4 are real diagrams

The IVP [15]: We first performed RC and extended pelvic lymph node dissection, and then constructed an anti-reflux neobladder. (1) Construction of the neobladder: A 48-cm segment of ileum was resected 15–20 cm from the ileocecal valve, and intestinal continuity was restored. The free intestinal segment was irrigated with 1% povidone iodine until it was clear. The dist 36–38 cm of the resected ileum was folded in half and arranged in a “U” shape, with the proximal 10–12 cm of ileum left as the input segment for anti-reflux and ureteral anastomosis. The mesentery of the folded intestinal segment longitudinally incised along the edge, and the inner edge of the U-shaped intestinal wall was continuously sutured with absorbable 3/0 vicryle form the posterior wall of the urinary pouch. After the U-shaped intestinal segment was folded again at the lowest part, the two outer edges of the intestinal segment were continuously sutured to form the urinary reservoir of the neobladder (Fig. 1A1-A2).(2) Construction of anti-reflux "ileal wall valve": The input intestinal segment was folded back and attached to the urinary reservoir, and input segment (1/2 circumference of the ileum) was sutured to the intestinal wall adjacent to the surface of the urinary reservoir from the junction of the port for about 2.5 cm, forming a rectangular (length × width = 2.5 cm × 1/2 of the ileal circumference)"flap-like" structure, namely, the anti-reflux "ileal wall valve" (Fig. 1B1-B2). A free intestinal segment of 8.0 cm in length was reserved for anastomosis with the left and right ureters.(3) “Drag-in” ureterointestinal anastomosis: The ureters on both sides were mobilized proximally about 5 cm, and the distal end was quickly frozen and sectioned. It is particularly important to note that: do not mobilize the retroperitone ureter (especially the left ureter) excessively, and try to keep it in its original position. Sometimes it can be transected close to the junction of ureter and the iliac vessels. A small hole was made on the back of the reserved input segment of the ileum, and the diameter of the hole was with the diameter of the ureter. The end of the ureter was dragged into the ileum about 1.0 cm with 4/0 or 5/0 vicryle absorbable double-needle thread (Fig. 1C1-C2), and the ureter was fixed to the seromuscular layer the ileum with two rows of 4–6 stitches (Fig. 1C3-C4), and the ureteral stent (using a single-sided pigtail tube of 6 or 7 French) was led out from the anterior wall of the neobladder through the anastomosis.(4) The neobladder was anastomosed with the urethra using 2/0 vicryle absorbable suture and a Fr 20–22 double-lumen balloon Foley catheter was placed for support, with 15 ml of water injected into the balloon. The capacity the neobladder was tested during the operation, generally reaching 100–130 ml, and any obvious leakage was repaired in a timely manner. stitches were used to suspend the anterior wall of the neobladder to the pubis. After hemostasis, 1–2 drainage tubes were placed laterally posteriorly to the neobladder. The abdominal incision was closed, and the operation was completed.MSP: A modified Studer neobladder technique was used [14, 16], and the urinary bladder was constructed in the same way as described above, with the proximal ileal input arm (about 12–15 cm) anastomosed to the ureter using the direct Nesbit technique.

Open or laparoscopic RC + ONB procedures were carried out, and both types of neobladders were constructed ex vivo(outside the abdominal cavity).

## Postoperative treatment and follow-up

### Postoperative management

The ureteral stent was removed at 12–14 days postoperatively and the catheter was removed at 19–21 days.

### Follow-up visits

The patients were followed up every 3 months in the first year, every 6 months in the second year, and annually in the third year after surgery. Follow-up visits included physical examination, assessment of voiding, urinary control, and sexual function [15, 19] (not discussed in this article), routine blood and urine tests, blood biochemistry (e.g., liver and kidney function), and urine culture. Abdominal ultrasonography was performed at each visit to assess UUT obstruction or effusion, and radiologic evaluation (e.g., CTU and MRU) was performed when necessary. The laboratory results, radiologic findings, and postoperative complications were recorded in detail, including those managed at other hospitals or physicians.

Events of interest included positive postoperative urine cultures (bacteriuria), complications, urinary tract infections that were symptomatic or required hospitalization, any surgical reoperation or urinary diversion-related reoperation (defined as open or endoscopic surgery under anesthesia), and survival. Complications were graded based on the modified Clavien-Dindo complication grading system [20]. Furthermore, renal function was assessed based on blood creatinine (normal values of 40.0–135.0 μmol/L) and the estimated glomerular filtration rate (eGFR) was calculated using the CKD-EPI (Chronic Kidney Disease Epidemiology Collaboration) equation [7, 21, 22]. renal function damage was defined as a decrease in eGFR value of > 10 ml/min/1.73 m^2^ following the surgery (postoperative eGFR—preoperative eGFR) [7, 23]. The primary study endpoint was overall survival time, with follow-up starting after surgery until the patient's death or final loss to follow-up.

### Statistical analysis

Data were statistically analyzed using the SPSS statistical package (IBM, version 27.0). Variables were compared between the two groups using the chi-square test, Mann–Whitney U test and Student's t-test. Survival curves such as cumulative caner-specific survival (CSS) and cumulative overall survival (OS) after treatment were determined using the Kaplan–Meier method, and the significance of differences was tested by log-rank test. Logistic regression model was established to analyze the correlates of renal function damage (postoperative eGFR-preoperative eGFR value > 10 ml/min/1.73 m^2^) [7, 23]. In this study, *p* < 0.05 was considered statistically significant.

## Results

### Comparison of clinical base

The clinical baseline characteristics of the two groups before and after PSM were similar and showed no significant difference (*p* > 0.05) (Table 1). A total of 84 cases (42 each for IVP and MSP) were successfully matched. Moreover, no statistically significant difference was observed between the two groups after matching 17 clinical factors, including comorbidities such as diabetes mellitus, preoperative eGFR, perioperative chemotherapy, and follow-up time (*p* > *0*.05).There was no significant statistical difference in the operation time(*p* = 0.128) and the time of urinary diversion (*p* = 0.354) between the two groups.

**Table 1:** Baseline characteristics of the type of urinary diversion before and after matching

Variables[Mean ± SD, or n (%)]	Before propensity score matching			After propensity score matching		
	IVP(*n* = 66)	MSP (*n* = 61)	*p*-value	IVP (*n* = 42)	MSP (*n* = 42)	*p*-value
Age (years)	64.50 ± 9.03	56.84 ± 6.90	0.353	65.40 ± 8.17	64.95 ± 6.89	0.784
Gender			0.790			0.776
Male	54 (81.8)	51(83.6)		34 (81.0)	35(83.3)	
Female	12 (18.2)	10 (16.4)		8 (19.0)	7 (16.7)	
BMI (kg/m^2^)	22.63 ± 2.57	23.18 ± 2.73	0.240	23.02 ± 2.64	22.85 ± 2.57	0.760
Smoking history			0.093			0.620
Never smoker	53 (80.3)	41 (67.2)		32 (76.2)	30 (71.4)	
Prior/Current smoker	13 (19.7)	20 (32.8)		10 (23.8)	12 (28.6)	
Associated comorbidities			0.490*			0.468*
Hypertension	15 (22.7)	16 (26.2)		10 (23.8)	12 (28.6)	
Diabetes	2(3.0)	5(8.2)		2(4.8)	0(0.0)	
Cardiac problems	1 (1.5)	2 (3.3)		1 (2.4)	0 (0.0)	
Preoperative Renal hydronephrosis/ureteral obstruction	11 (16.7)	8 (13.1)	0.575	4(9.50)	6(14.3)	0.500
Preoperative eGFR(ml/min/1.73 m^2^)	85.21 ± 19.20	85.95 ± 18.23	0.825	87.43 ± 19.15	88.02 ± 18.81	0.886
ASA Physical Status			0.710*			1.000*
≤ 2	63 (95.5)	57 (93.4)		40 (95.2)	41 (97.6)	
> 3	3 (4.50)	4 (6.60)		2 (4.80)	1 (2.4)	
Surgical approach			0.662			0.724
Open surgery	7(10.6)	8 (13.1)		5 (11.9)	4 (9.5)	
Laparoscopic surgery	59 (89.4)	53 (86.9)		37 (88.1)	38 (90.5)	
Nerve-sparing	57(86.4)	53 (86.9)	0.931	38 (90.5)	36(85.7)	0.500
Operating time	359.4 ± 41.27	347.30 ± 48.32	0.128	361.0 ± 40.50	350.31 ± 50.50	0.284
Urinary diversion time	42.88 ± 9.37	40.90 ± 7.61	0.196	42.74 ± 9.50	40.95 ± 8.28	0.354
Pathologic T stage						0.794
T1-2	50 (75.8)	45(73.8)	0.797	33 (78.6)	32 (76.2)	
T3-4	16 (24.2)	16 (26.2)		9 (21.4)	10 (23.8)	
Postoperative grade			0.783*			0.717*
G1	4(6.10)	3 (4.90)		1(2.4)	3(7.1)	
G2	8 (12.1)	10 (16.40)		4 (9.5)	4(9.5)	
G3	54 (81.8)	48 (78.70)		37 (88.1)	35 (83.3)	
Number of lymph nodes	15.98 ± 3.89	15.82 ± 3.39	0.800	15.83 ± 3.44	15.48 ± 2.74	0.600
Combined carcinoma in situ	7 (10.6)	3 (4.9)	0.328*	2(4.8)	3(7.1)	1.000*
Abnormal pathology (adenocarcinoma/squamous cell carcinoma, etc.)	14(21.2)	7(11.5)	0.140	7(16.7)	4(9.5)	0.332
Neoadjuvant chemotherapy	11(16.7)	6(9.8)	0.259	5 (11.9)	6 (14.3)	0.746
Follow-up time [M(IQR),months]	44.5(22.5–54)	39(21.5–56.5)	0.927**	44.5(25–55)	35.5(20–50)	0.447**

*Abbreviations: VIP* ileum valve- pouch, *MSP* Modified Studer- pouch, *BMI* Body mass index, *eGFR* Estimated glomerular filtration rate, *ASA* American Society of Anesthesiologists. *M* median, *IQR* Interquartile range

^*^ Fisher’s exact test

^**^ Mann–Whitney U-test

### Oncologic findings

**Fig. 2:**
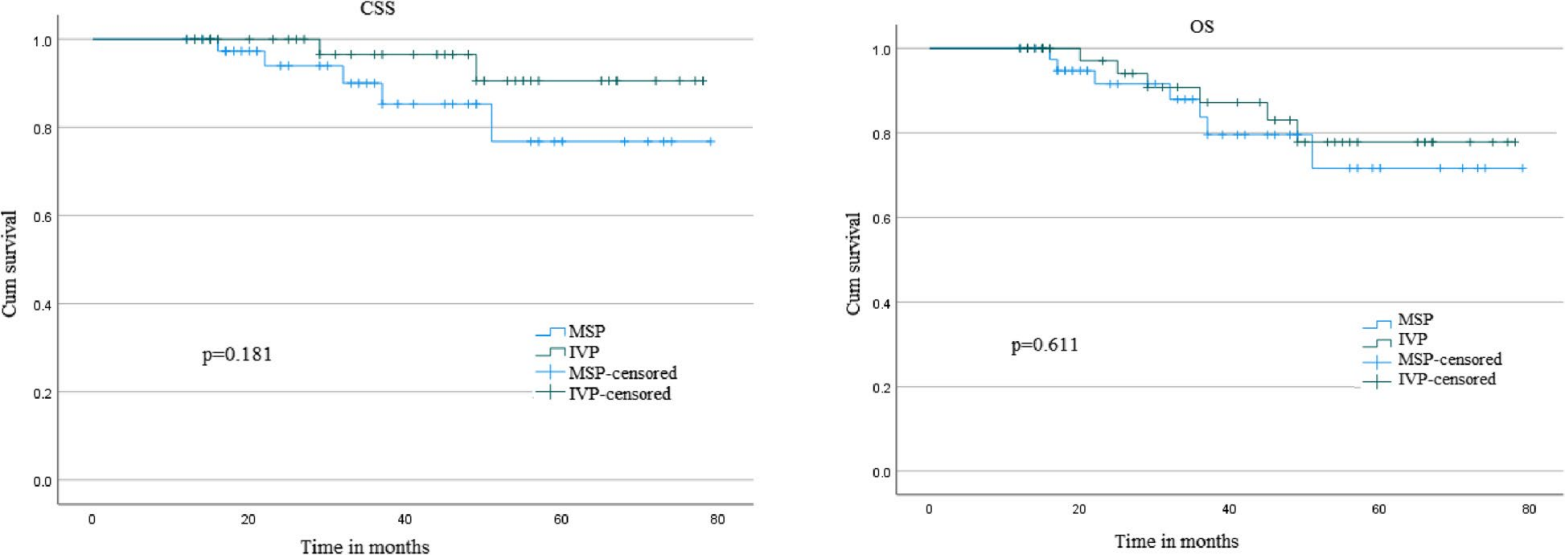
Kaplan–Meier curves. **A** cancer-specific survival(CSS), **B** overall survival(OS). Abbreviations: IVP:ileum valve- pouch,MSP:modified Studer- pouch

The median follow-up times were 44.5 (25-55) and 35.5 (20-50) months in the IVP and MSP groups, respectively. In the IVP group, the 3-year and 5-year CSS were 96.6% and 90.5% respectively; in the MSP group, the 3-year and 5-year CSS were 85.3% and 76.8%, respectively (Figure 2A, *p*=0.181). In the IVP group, the 3-year and 5-year OS were 87.2% and 77.8%, respectively; in the MSP group, the 3-year and 5-year OS were 83.7% and 71.6%, respectively (Figure 2B, *p*=0.611). The two groups showed no statistically significant differences in the survival curves (p>0.05) (Figure 2).

### Surgical complications

No perioperative deaths or intraoperative complications were found in both the IVP and MSP groups. Nonetheless, three cases of Clavien-Dindo grade III (one each of incisional split, intestinal fistula, and ischemic intestinal obstruction) occurred in the MSP group within 30 days of surgery. Re-hospitalization due to urinary tract infection demonstrated statistically significant difference between the two groups, with 10 (23.8%) cases in the IVP group and 19 (45.2%) patients in the MSP group (*p* = 0.039). However, other complications showed no significant difference between the two groups (*p* > 0.05) (Table 2).

**Table 2:** Postoperative complications in the type of urinary diversion after matching

Variables[ n (%)]	IVP (*n* = 42)	MSP (*n* = 42)	*X*^*2*^	*p*-value
Postoperative complications (Clavien-Dindo grade)				0.173^*^
I	12(28.6)	9 (21.4)		
II	9(21.4)	14(33.3)		
III	0 (0.0)	3(7.1)		
Re-hospitalization for urinary tract infections, etc	10 (23.8)	19 (45.2)	4.266	0.039
Urinary retention requiring catheterization /recurrent bladder stones after surgery	3(7.1)	4(9.5)		1.000*
Postoperative upper urinary tract obstruction/hydronephrosis	5(11.9)	7(16.7)	0.389	0.533
Simple renal hydronephrosis	4(9.5)	0(0.0)		0.116*
Hydronephrosis with ureteral dilation	1(2.4)	5(11.9)		0.202*
Anastomotic stricture requiring surgery	0(0.0)	2(4.8)		0.494*

*Abbreviations: VIP* ileum valve- pouch, *MSP* Modified Studer- pouch

^*^ Fisher's exact test

### Postoperative renal function changes after matching

At 6 months postoperatively, there were no statistically significant differences between the two groups except for the case of abnormal creatinine (> 135 μmol/L) (*p* = 0.012) before matching; after, there were no differences between the two groups (*p* > 0.05). At 12 months postoperatively, there were statistically significant differences between the two groups before matching (*p* < 0.05); after matching, there were differences in eGFR values (*p* = 0.031) and renal function damage (*p* < 0.001), but not in the rest(*p* > 0.05) (Table 3).

**Table 3:** Renal function outcomes of the type of urinary diversion before and after matching

Variables[Mean ± SD, or n (%)]	Before propensity score matching			After propensity score matching		
	IVP(*n* = 66)	MSP (*n* = 61)	*p*-value	IVP (*n* = 42)	MSP (*n* = 42)	*p*-value
Postoperative 6 months						
Serum creatinine level( μmol/L)	101.16 ± 59.40	106.46 ± 50.81	0.592	105.90 ± 72.31	103.25 ± 48.31	0.844
Abnormal creatinine(> 135 μmol/L)	4(6.1)	13 (21.3)	0.012	3(7.1)	6 (14.3)	0.483
eGFR value(ml/min/1.73 m^2^)	74.65 ± 20.80	70.86 ± 26.49	0.370	74.12 ± 22.79	72.51 ± 25.50	0.762
Renal function damage^a^	31(47.0)	38 (62.3)	0.083	23(54.8)	26 (61.9)	0.507
Postoperative 12 months						
Serum creatinine level( μmol/L)	96.76 ± 39.70	131.33 ± 107.19	0.016	95.83 ± 48.31	136.16 ± 126.42	0.051
Abnormal creatinine(> 135 μmol/L)	6(9.1)	15 (24.6)	0.019	5(11.9)	10 (23.8)	0.154
eGFR value(ml/min/1.73 m^2^)	76.75 ± 26.32	60.23 ± 25.38	< 0.001	75.93 ± 26.53	61.03 ± 26.89	0.013
Renal function damage^a^	27(40.9)	48 (78.7)	< 0.001	19(45.2)	34 (81.0)	< 0.001

*Abbreviations: VIP* ileum valve- pouch, *MSP* Modified Studer- pouch, *eGFR* Estimated glomerular filtration rate

^a^Renal function damage:postoperative eGFR-preoperative eGFR > 10 ml/min/1.73 m^2^

### Analysis of factors related to renal function damage

Logistic regression analysis was performed to identify factors associated with renal function damage at 12 months postoperatively. Univariate analysis revealed that new bladder type (*p* = 0.001), preoperative eGFR value (*p* < 0.001), re-admission for urinary tract infection (*p* = 0.003), and positive postoperative urine culture (bacteriuria) (*p* = 0.002) were significantly associated with renal function damage at 12 months postoperatively. The multivariate analysis indicated that neobladder type (*p* = 0.004) and preoperative eGFR value (*p* < 0.001) were significantly associated with renal function damage at 12 months postoperatively (Table 4).

**Table 4:** Analysis of factors related to renal function damage after matching

Variables	univariate analysis		multivariate analysis	
	OR (95% CI)	*p*-value	OR (95% CI)	*p*-value
Type of urinary diversion ( VIP/MSP)	5.145 (1.929–13.722)	0.001	6.172 (1.764–21.590)	0.004
Preoperative eGFR (Mean ± SD,ml/min/1.73 m^2^)	1.055 (1.024–1.086)	< 0.001	1.077 (1.036–1.119)	< 0.001
Re-hospitalization for urinary tract infections, etc. (n, %)	0.116(0.051–0.540)	0.003	0.115(0.007–1.925)	0.132
Positive postoperative urine culture (bacteriuria) (n, %)	0.154 (0.047–0.501)	0.002	0.889 (0.063–12.250)	0.930

*Abbreviations: VIP* ileum valve-pouch, *MSP* Modified Studer-pouch, *eGFR* estimated glomerular filtration rate

## Discussion

ONB is physiologically close to normal bladder and improves quality of life. With the advancement of robotic instruments and surgical techniques, ONB has been increasingly explored by scholars [1, 2, 24, 25]. UEA is a pivotal step in the ONB construction process to protect the UUT. Currently, two techniques are primarily used, including the reflux and anti-reflux techniques. However, the necessity of AR anastomosis remains controversial [4, 10–12]. Drawing on the technical experience of general surgery bile-intestinal AR and pancreatic-intestinal sleeve-in anastomosis, the author created the anti-reflux ileum valvuloplasty and ureteral drag into ileal anastomosis technique.This technique can prevent both the reflux of urine from the neobladder to the UUT and EUA stenosis, and better protect UUT and renal function.

Ileum valve AR mechanism (1) the smooth peristaltic ileum and detubularized distal part of the pouch are tightly sutured, the AR effect is mediated by the peristaltic movement of the attached ileum, driven by the synchronization of smooth peristaltic movement. (2) The folded part of the pouch bridge flap surface and the attached ileum wall form a live flap that could be swung back and forth, producing the AR effect. (3) The short-armed ileum and the pouch were sutured in parallel for 2.5 cm, and the urine in the ileum was injected into the pouch at an angle of almost 180 degrees to form a unidirectional open "rectangular flap" (Fig. 1B), preventing urine reflux. (4) The use of a peristaltic ileal arm of only 10–12 cm, without the need for 20–25 cm [13], preserves the lower arm ureter with its intrinsic AR properties [20]. In addition, the morphology, volume and pressure of this neobladder are very close to the natural bladder [15, 19].

The choice of UEA anastomosis remains highly controversial [2–8, 11, 12]. AR anastomosis is associated with a higher rate of anastomotic stricture. Among various anastomotic techniques, the anastomotic stricture rate of AR anastomosis is 2% to 29%,which is twice as high as that of direct anastomosis [8, 24–27]. No cases of anastomotic stenosis or regurgitation were seen in our group, and complications such as anastomotic fistula were avoided. The ureter was dragged into the ileocecal anastomosis based on the concept of pancreatic-intestinal sleeve-in anastomosis in general surgery (Fig. 1C). Specifically, the ureter was dragged 1 cm into the ileum of the input arm of the neobladder and fixed to the intestinal wall at an angle of 90 degrees, yielding a living flap-like AR effect. (2) Both the right and left ureters were anastomosed to the posterior wall of the ileal arm near the distal end, without the need to free the ureter for too long, so that both sides of the ureter maintain a natural anatomical position, preserving its own and surrounding blood supply. (3) It is not necessary to free and displace the ureter extensively, nor is it necessary for the left ureter to cross the sigmoid mesentery to the right side to avoid periureteral fibrosis or angulation. The results of the present study revealed that this anti-reflux technique could avoid UEA stenosis, in accordance with previously published results [15, 19].

The most critical aspect of UEA anastomosis is the protection of UUT and renal function. Anastomotic stenosis is a major risk factor for renal impairment and is difficult to manage. The exact cause is not known [28–30]. Recently, Benson CR et al. reported that it is closely related to UUT infection [28]. The presence of reflux does not significantly alter renal function [8, 28]. Therefore, a growing number of scholars recommend reflux anastomosis [6–9]. Postoperative renal function damage is influenced by multiple factors such as age, preoperative eGFR, comorbidities such as hypertension and diabetes mellitus, tumor status, perioperative chemotherapy, sepsis, UUT obstruction, etc. [21, 22]. Skinner et al. [7] conducted a randomized trial comparing Studer-pouch and T-pouch in ONB, revealing that despite the AR mechanism of the T-pouch, a moderate decline in renal function was observed at 3 years compared with Studer-pouch. The deterioration in renal function was associated with age, preoperative eGFR, and urinary tract obstruction. The long construction time and the need to displace the ureter due to the complexity of the T-pouch anti-reflux maneuver are the main reasons for the increase in secondary procedures.In addition, the study did not include key factors affecting renal function, such as hypertension and diabetes, in the analysis, which may have some impact the objectivity and accuracy of the renal function damage after surgery.In this study, PSM was performed to minimize confounding factors between groups by including all 17 relevant factors (including comorbidities such as hypertension and diabetes mellitus) that may affect the renal function damage. The IVP group significantly reduced the 1-year postoperative renal function damage rate compared with the MSP group. Renal function damage was associated only with neobladder type and preoperative eGFR.

Advantages of VIP technique: The "intestinal wall valve" of VIP provides a more reliable anti-reflux mechanism. VIP-Pouch requires a short of ileum and has a good functional effect (the preliminary and intermediate results are satisfactory, and the long-term efficacy is still to be determined). It almost does not the additional time for constructing the anti-reflux mechanism (about 2 min). At the same time, it can reduce the re-admission rate and the rate of (within 12 months after surgery) renal function damage (decrease). However, there are still cases of anastomotic stenosis (2 cases 4.8%) that require reoperation in the MSP group.

## Limitations

Firstly, this study was a retrospective study with a small sample size, lack of comparison with other direct AR anastomotic techniques and incomplete follow-up indicators(such as the lack of residual urine determination and the limited scope of investigation application). However, the early and mid-term functional outcomes and the incidence of postoperative complications associated with the technique are acceptable, and the trend of the parameters of the recent renal function changes after RC seems to be superior to that of the Studer technique. Future studies should further expand the number of patients and the scope of application, and adopt a randomized design to overcome the existing limitations.Second, The statistical sample data between the two groups were not perfectly balanced, and there was a selection bias. Although we performed a propensity score matching analysis for relevant influencing factors (17 items) to minimize the bias, a prospective randomized controlled trial is still the best. Meanwhile, the operation skills of different surgeons, the conditions and environment of the hospital, etc., may also affect the surgical outcomes. Therefore, all surgeries this study were performed or participated in by a senior attending surgeon (ZHU) with rich experience under supervision to minimize their effects.Third, it is unclear whether the eGFR can accurately assess renal function in patients with an ileal neobladder (because the ileum-constructed reservoir will absorb creatinine and urea nitrogen from the urine and affect renal function), but the length of the ileum used for neobladder construction was similar the two groups to minimize the bias of renal function data between the groups.Finally, since most of the patients come from rural areas, compliance is poor.After more than one year of surgery, when the patients' condition recovers well, at each follow-up time point, a small part of patients ( about 17%) did not return to the hospital for examination and follow-up, resulting in lack of data, such as renal function.In the future, it is necessary to focus on the long-term (over 3 years) changes in renal function, complications, and secondary surgery.And further prospective, randomized controlled trials are needed to confirm its efficacy.The content has been modified in the paper.

## Conclusion

The preliminary results of VIP technique were safe and effective, which did not significantly increase the time of anti-reflux construction (2 min), and the incidence complications was similar in the two groups, but the protection of renal function at 12 months after surgery seemed to be better than MSP.The independent factors affecting renal function damage are neobladder type and preoperative eGFR. However, longer-term follow-up and randomized studies are required to further evaluate the advantages and disadvantages of this technique.

## Data Availability

Data is provided within the manuscript or supplementary information files. The datasets generated and analysed during the current study are not publicly available due to do not have consent from all patients to publish this data, but are available from the corresponding author on reasonable request.
